# Climate change and mammals: evolutionary versus plastic responses

**DOI:** 10.1111/eva.12121

**Published:** 2013-12-13

**Authors:** Stan Boutin, Jeffrey E Lane

**Affiliations:** Department of Biological Sciences, University of AlbertaEdmonton, AB, Canada

**Keywords:** adaptation, climate change, contemporary evolution, ecological genetics, natural selection, phenotypic plasticity, quantitative genetics

## Abstract

Phenotypic plasticity and microevolution are the two primary means by which organisms respond adaptively to local conditions. While these mechanisms are not mutually exclusive, their relative magnitudes will influence both the rate of, and ability to sustain, phenotypic responses to climate change. We review accounts of recent phenotypic changes in wild mammal populations with the purpose of critically evaluating the following: (i) whether climate change has been identified as the causal mechanism producing the observed change; (ii) whether the change is adaptive; and (iii) the relative influences of evolution and/or phenotypic plasticity underlying the change. The available data for mammals are scant. We found twelve studies that report changes in phenology, body weight or litter size. In all cases, the observed response was primarily due to plasticity. Only one study (of advancing parturition dates in American red squirrels) provided convincing evidence of contemporary evolution. Subsequently, however, climate change has been shown to not be the causal mechanism underlying this shift. We also summarize studies that have shown evolutionary potential (i.e. the trait is heritable and/or under selection) in traits with putative associations with climate change and discuss future directions that need to be undertaken before a conclusive demonstration of plastic or evolutionary responses to climate change in wild mammals can be made.

## Introduction

There can be little doubt that climate has acted as a strong selective force in the past, but rapid contemporary climate change is affording evolutionary ecologists the opportunity to study its influence first hand. The scientific excitement surrounding this opportunity has prompted many to re-examine their long-term records and the number of reported cases of climate-driven phenotypic changes has grown rapidly since the mid-eighties (Parmesan 2006 and references therein). However, what is the actual evidence that these changes reflect climate-driven evolution in action? Separating the climate signal from other contemporary factors as the causative agent is not straightforward given the correlation between changing climate and other human activities (Millenium Ecosystem Assessment 2005) and the difficulty in creating “experimental climates”. In addition, clearly not all phenotypic change is due to evolution and as Merilä and Hendry (2014) point out, separating the relative influences of evolution and plasticity underlying phenotypic change is extremely challenging. In most cases, long-term continuous records of phenotypic traits in populations are now just reaching 20–30 years in duration. Although climate is considered to be changing rapidly, is this length of time sufficient to observe climate-driven evolution?

To date, the majority of examples of climate-driven phenotypic changes in plants and animals have been phenological in nature. Warming temperatures in temperate and polar regions, for example, are shifting growing seasons which has the potential to be a strong selective force on the timing of spring migration, termination of hibernation and reproduction (Forrest and Miller-Rushing 2010). Other possible changes could involve changes in body size (Bergmann's Rule predicts smaller body size with increased temperature whereas increased ecosystem productivity could lead to larger body size (Yom-Tov and Yom-Tov 2004; Teplitsky and Millien 2014) and possibly even metabolic rates, should variation across time resemble that across space (i.e. zoogeographical zones; Lovegrove 2000). Still other changes, such as those brought about by the selective forces of new competitors, predators, parasites and diseases will surely follow but, for the most part, remain undocumented. Vertebrate examples of phenological changes brought on by climate change involve primarily birds (Møller et al. 2010). Examples in mammals are fewer but important because, unlike many temperate and polar bird species studied to date, most mammal species in these regions are sedentary requiring *in situ* seasonal adaptations such as hibernation or the storing of energy as fat or food caches to cope with long periods of resource shortage. Furthermore, large-bodied mammals have long gestation periods so breeding has to be timed to link high quality resources with lactation demands months into the future. From a conservation standpoint, developing a better appreciation of the adaptive potential of mammals is important because, due to their limited dispersal ability relative to birds, range shifts of many mammalian species may be unable to track the pace of rapid climate change (Schloss et al. 2012).

Phenotypic plasticity affords a relatively rapid adjustment of life history and morphological traits to environmental variation. It could thus be an important process of short-term adaptation. However, as the phenologies of trophic levels may respond differently to climate change (sensu Thackeray et al. 2010) current levels of plasticity may no longer be fully adaptive. Moreover, consensus projections from current climate models are for long-term directional change (IPCC 2007). As phenotypic plasticity has a theoretical limit, it is thus assumed that microevolutionary change will be necessary to prevent population declines (Lande and Shannon 1996; Phillimore et al. 2010). We review the documented phenotypic changes observed in mammals that have been assumed to be associated with recent climate change, and assess the evidence that these changes are due to climate rather than some other environmental factor, that the observed changes are adaptive, and that the changes are a result of phenotypic plasticity and/or evolution.

## Phenotypic changes in mammals and their link to climate change

We begin by reviewing the evidence for a causative link between climate change and phenotypic changes in mammals. We focused on the literature cited in recent papers and expanded our search through the Web of Science (using the following combinations of key words; climate change + mammal, climate warming + mammal, global warming + mammal). We included only those studies involving recent (within the last century) climate and phenotypic changes. Our search produced a total of 19 studies, 12 involved measurements on live mammals, while seven measured changes in body size using museum specimens (Table 1). Of the live animal studies, traits measured included the following: reproductive timing (six studies), emergence from hibernation (three studies), body mass (five studies) and litter size (one study) and involved 11 herbivores (three small, four mid-size (all hibernators), and four large (all ungulates) species) and one carnivore. The size range among the species was <100 g to >500 kg. Study duration ranged from 6 to 60 years with the average being 23 years. The museum studies measured body size changes of various species (primarily carnivores) collected over the last 40–60 years. The results of these studies will be briefly summarized below but because they are thoroughly covered by Teplitsky and Millien (2014), we do not consider them further.

**Table 1:** Mammalian studies that have assessed change in phenotypic traits in response to climate change

Species	Trait	Genetic	Plastic	Adapt	Cause	Driver	Years	References
Small mammals								
*Peromyscus maniculatus*	BD	.	N(2)	.	N(2)	TP	18	Millar and Herdman (2004)
*Tamiasciurus hudsonicus*	BD	Y(1,2)	Y(1,2,3)	Y(1)	Y(2)	TP, F	10	Réale et al. (2003a)
*Neotoma albigula*	BS	.	.	.	Y(2)	TP	8	Smith et al. (1998)
Hibernators								
*Marmota flaviventris*	BD, BS, HT	N(1)	Y(1)	Y(1,2)	Y(1)	TP, S	33	Ozgul et al. (2010); Inouye et al. (2000)
*Marmota marmota*	LS,BS	.	Y(2)	N(2)	Y(2)	TP, S	20	Tafani et al. (2013)
*Urocitellus columbianus*	HT	.	Y(2,3)	N(1,2)	Y(2)	S	20	Lane et al. (2012)
*Glis glis*	HT	.	.	.	Y(2)	TP	26	Adamik and Kral (2008)
Ungulates								
*Cervus elaphus*	OD,BD,AC, RS,RE	.	Y(1,2,3)	N(1,2)	Y(2)	GDD	28	Moyes et al. (2011)
*Ovis aries*	BS	N(1)	Y(1,2,3)	Y(2)	Y(2)	NAO	20	Ozgul et al. (2009)
*Bos taurus*	BD	.	Y(2)	N(2)	Y(2)	TP,NAO,GDD	60	Burthe et al. (2011)
*Rangifer tarandus*	BD	.	Y(2)	N(2)	Y(2)	SGS	6	Post and Forchhammer (2008)
Carnivores								
*Ursus maritimus*	BS	.	Y(2)	N(2)	Y(2)	SI	28	Stirling and Derocher (2012)
Museum studies								
*Sorex cinereus*	BS	.	.	.	.	TP	53	Yom-Tov and Yom-Tov (2005)
*Martes americana*	BS	.	.	.	Y	TP	49	Yom-Tov et al. (2008)
*Lutra lutra*	BS	.	.	.	N	TP	30	Yom-Tov et al. (2006)
*Vulpes vulpes*	BS	.	.	.	N	TP	26	Yom-Tov et al. (2007)
*Two rodents*	BS		.		N	TP	69	Yom-Tov and Yom-Tov (2004)
*22 carnivores*	BS		.		.	TP	40	Meiri et al. (2009)
*Five carnivores*	BS	.	.	.	N	TP	50	Yom-Tov (2003)

*Trait* (type of trait examined): BD, Birth date; OD, Oestrus date; AC, antler cast or Antler Cleaning; RS, start of rut; RE, end of rut; BS, Body size or mass; HT, Hibernation termination; LS=Litter size. A ‘Y’ in respective columns indicates that evidence was found for Genetic or Plastic responses in traits, the response was adaptive (Adapt), and climate change was the causative agent (Cause); ‘N’ indicates evidence was not found for Genetic or Plastic responses in traits, the response was not adaptive, and the causative agent was not climate change; ‘.’ indicates that it was not investigated. Numbers next to a ‘Y’ or ‘N’ denote the method of investigation invoked, in cases with no numbers, a method was invoked that does not fit into one of the categories used for this review. *Genetic categories*: 1: Quantitative genetics; 2: Comparison to model predictions. *Plastic categories*: 1: Quantitative genetics; 2: Fine-grained population responses; 3: Individual plasticity in nature. *Adapt categories*: 1: Phenotypic selection estimates; 2: Changes in the phenotypic trait were in the direction that would increase fitness but relative fitness was not measured. *Cause categories*: 1: Common sense; 2: Phenotype by environment interactions. For full descriptions of all categories see Merilä and Hendry (2014). *Driver* (causal driver of change): NS, not specific; TP, temperature; PR, precipitation; S, snow melt or snow depth; F, food; SI, sea ice break-up; NOA, North Atlantic Oscillation; GDD, Growing degree days; SGS, start of growing season. *Years* (length of study).

### Reproductive timing

**Figure 1:**
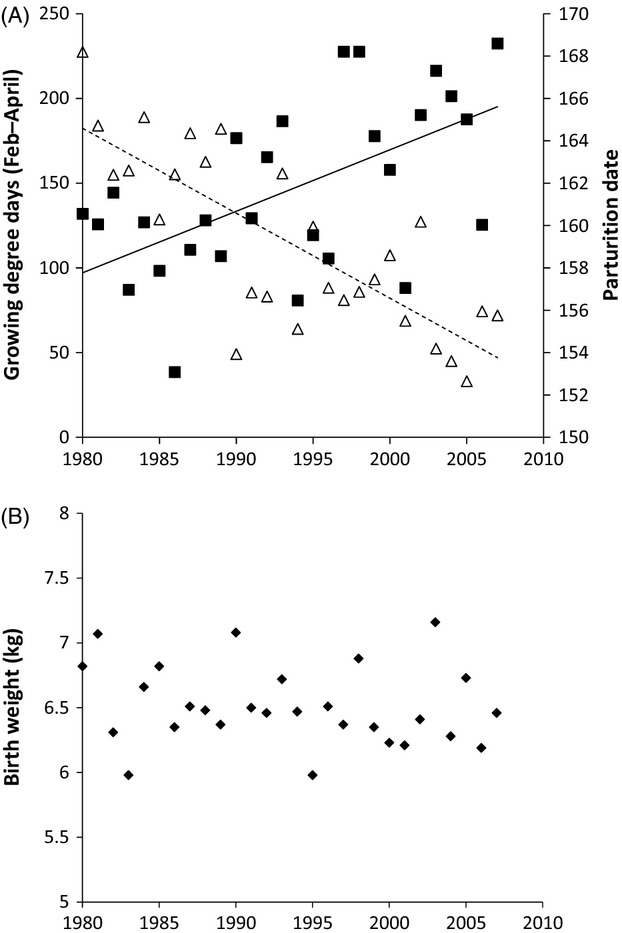
An example of advances in breeding dates in mammals with warming temperatures. Moyes et al. (2011) found that parturition date (Julian date, triangles and dotted line) has advanced in red deer as the number of Growing Degree Days (squares and solid line) has increased (A). The advances in breeding have not been associated with an increase in fitness components; offspring birth weight is provided as an example (B). Figures redrawn from Figs 1, 2A and 3A in Moyes et al. 2011).

Moyes et al. (2011) present changes in six phenological traits in red deer (*Cervus elaphus*) on the Isle of Rum, Scotland over a 28-year period: oestrous date and parturition date in females (see also Coulson et al. 2003), and antler cast date, antler clean date, rut start date and rut end date in males. In all cases, the dates have advanced by 5–12 days and these changes were associated with increases in the number of growing degree days (i.e. the sum of daily average temperatures above a threshold across a set period of time; Moyes et al. 2011; see Fig. 1 for an example). Post and Forchhammer (2008) found that calving date in caribou (*Rangifer tarandus*) on Greenland advanced 3.82 days (0.29 day year^−1^), while the onset of plant growth (driven by warming temperature) advanced 4.59 days (0.35 day year^−1^) over a 13-year period. Continuous measurements were available in the last 5 years of the study only and the authors argue that the magnitude of change in plant growth over this time was much greater than the change in calving date, creating a trophic mismatch. Burthe et al. (2011) document advances in birth dates of roughly 2 months in Chillingham cattle (*Bos taurus*) over a 60-year period (1.00 day year^−1^) in northeast UK. This population has lived in a small enclosure in a feral state (hay is provided each winter) since the 16th century and calves can be born at any time of the year. The advancement of timing of breeding has led to a higher proportion of births occurring in the winter months with this proportion being positively related to warmer temperatures and earlier initiation of the growing season during the spring of conceptions.

Millar and Herdman (2004) found no change in first parturition dates for deer mice (*Peromyscus maniculatus*) over an 18-year period in Alberta, Canada. In this area, spring temperatures actually cooled by 2°C leading to an 11-day delay in the date when average temperature went above freezing between the start and end of the study. Ozgul et al. (2010) report a 5.6-day advance in weaning date of yellow-bellied marmots (*Marmota flaviventris*) in Colorado, USA over a 33-year period (0.17 day year^−1^), but they do not establish a link between these changes and climate. We have studied American red squirrels (*Tamiasciurus hudsonicus*) in Yukon, Canada since 1989 and during the first 10 years of study we found that first parturition dates advanced by 18 days (1.80 day year^−1^; Réale et al. 2003a). In our system, red squirrels larderhoard white spruce (*Picea glauca*) cones in the autumn and use the seed from these cones to survive over winter and to fuel reproduction in the following spring. Spruce is a masting species and autumn cone availability can vary by 2 orders of magnitude (Boutin et al. 2006; LaMontagne and Boutin 2007). Average parturition date of squirrels is highly variable from year to year (range of annual means: April 2 to June 6) and inversely correlated with cone production in the previous autumn (higher production-earlier breeding; Boutin et al. 2006). Réale et al. (2003a) suggested that warmer spring temperatures and drier conditions due to climate warming could increase the likelihood of large mast years and thus affect parturition date. The evidence for the link between climate change and cone production was weak and 10 more years of data suggest that the advancement in parturition date observed in the first 10 years of study was unlikely to be due to climate change. Rather the intermast interval is relatively long (∼ 5+ years), relative to the study duration, and the advance was more likely due to natural underlying fluctuations in the cone crops unrelated to changing climate (J. E. Lane and S. Boutin, unpublished manuscript).

### Emergence from hibernation

**Figure 2:**
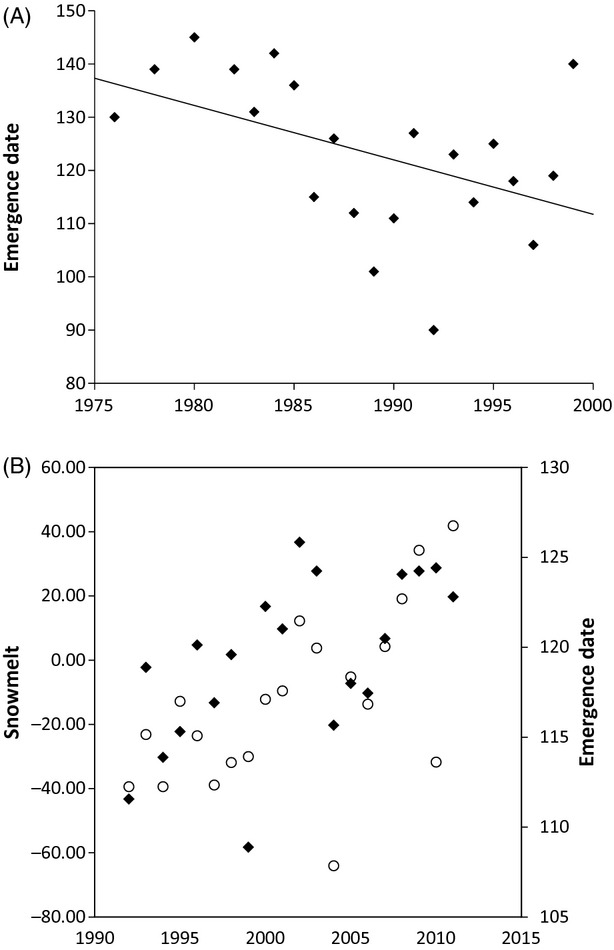
Contrasting changes in emergence date of two hibernators in the Rockies of North America; yellow-bellied marmots have advanced emergence (A), whereas Columbian ground squirrels have delayed emergence (B, Julian days open symbols), the latter in response to later snow melt (standardized values shown, open symbols). Redrawn from Fig. 4 of Inouye et al. (2000) and Fig. 1 of Lane et al. (2012).

Adamik and Kral (2008) found that edible dormice (*Glis glis*) advanced their date of termination of hibernation by approximately 19 days over a 25-year period (0.77 day year^−1^) in the Czech Republic and this was associated with an increase in mean monthly temperatures during April to June. Similarly, yellow-bellied marmots were observed to emerge 23 days earlier from 1976 to 1999 (1.00 day year^−1^); Inouye et al. 2000; Fig. 2A). Emergence date was highly correlated with April temperature each year but there was no significant increase in April temperature or date of initiation of the growing season over the course of the study (Inouye et al. 2000). Despite this, the authors still inferred that the trend in emergence date was related to warmer springs. In contrast, Lane et al. (2012) report that Columbian ground squirrels (*Urocitellus columbianus*) emerged from hibernation 9.4 days later over a 20-year time span (0.47 day year^−1^) in Alberta, Canada. This unusual change in timing was associated with an increased frequency of spring snow storms and later average snowmelt (Fig. 2B).

### Body mass and litter size

**Figure 3:**
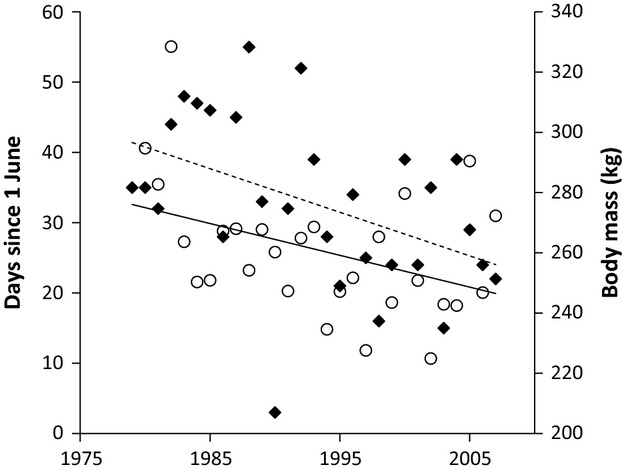
An example of changes in body mass of mammals in response to climate change. Polar bears have decreased in body weight (open symbols, solid line) as the timing of sea ice melt (Julian date, closed symbols, dotted line) has advanced. Redrawn from Figs 1 and 3, Stirling and Derocher (2012).

In the yellow-bellied marmots reported previously, body mass prior to hibernation increased roughly 10% over the 33-year study due to earlier emergence from the previous hibernation (Inouye et al. 2000), earlier weaning and a longer active season (Ozgul et al. 2010). The changes, although relatively gradual, have been accompanied by rapid increases in population size since 2000 but the link to climate change remains inferential (e.g. see Inouye et al. 2000). In contrast to yellow-bellied marmots, female alpine marmots (*Marmota alpinus*) in the French Alps have shown a decrease in body mass since 1990 and this is leading to smaller litters (Tafani et al. 2013). These decreases are correlated with a decreasing winter snow pack. Smith et al. (1998) documented a decline in body mass of white-throated wood rats (*Neotoma albigula*) in New Mexico, USA. Temperatures warmed over the course of the 8-year study and for every one degree increase in temperature there was a 10 g decrease in body weight. Soay sheep (*Ovis aries*) on St. Kilda, UK, have also shown a trend of decreasing body mass over a 20-year period. The argument for this is that changing climate has led to fewer harsh winters and longer growing seasons allowing for slower growing sheep to survive over-winter. This has led to lower overall average growth rates of individuals. Finally, Stirling and Derocher (2012) summarize the evidence for effects of changing climate on sea ice conditions and polar bear (*Ursus maritimus*) body mass (Fig. 3). They report a trend of decreasing body condition index and mass of polar bears in Hudson's Bay over a 28-year period, and this is strongly correlated with earlier timing of sea ice break-up (Fig. 3), which in turn reduces the time bears have to hunt seals. To summarize, four of the five studies of changes in body mass have documented declines with climate warming. The one exception, yellow-bellied marmots, showed an increase, but although climate warming is inferred as the causative mechanism, there has been no phenotype by environment correlational analyses to support this contention.

We found an additional seven museum studies that looked for trends in body size of mammals (mostly carnivores) over the last half of the 20th century (Table 1). The simple rationale behind these studies was that changing climate should lead to changes in body size as predicted by Bergmann's Rule. In six different papers by Yom-Tov and colleagues (see Table 1 for references), body size was found to increase in all of the species studied with the suggested causal mechanism being increased food supply or decreased energetic demands. However, in only three of these do the authors make a case for the hypothesized change being linked to climate change. Meiri et al. (2009) measured body size in 22 species of carnivores from 52 different populations. They found body size changes in only six cases with half showing an increase and half showing a decrease. Thus overall, the museum studies provide only limited support for climate change affecting body size of mammalian carnivores over the last half of the 20th century.

To summarize, of the 12 studies we found that assessed whether changes in climate have been associated with phenotypic change in live mammals, nine show relatively strong phenotype-environment correlations, two made “common sense” arguments and one paper found no relationship between trait and climate variation. The studies report warming temperatures leading to advanced and/or lengthened growing seasons or earlier sea ice melt with one exception that reports a delay in seasonal timing. Breeding phenology changed in the same direction as the climate, while body mass showed a pattern of decline in four of five cases. Museum studies found mixed evidence for changing body size in recent times but in most cases where changes did occur, body size increased. However, the causal link to climate tended to be weak to nonexistent.

## Are phenotypic responses adaptive?

Determining whether a phenotypic response to climate change is adaptive is not intuitive. If we take an advancing phenology in a temperate environment as an example, on one hand earlier breeding is often favoured because, among other reasons, it affords the young of the year a longer period of development before winter (e.g. Ozgul et al. 2010). A phenological advance can thus be considered an adaptive response. On the other hand, if the primary food source of the population is advancing at a greater rate than the population itself, individuals are likely to suffer fitness declines. In addition, the selective agent needs to be identified. Is it that earlier breeding relative to an environmental variable is favoured, or is it relative to conspecifics? We thus deem a phenotypic response as adaptive if fitness in the population is maintained following climate change. Such an adaptive response can occur if each individual responds so as to maintain their level of fitness (i.e. adaptation through phenotypic plasticity) or if individuals with certain genotypes (e.g. earlier breeders) experience higher fitness and thus contribute a greater proportion of surviving young (i.e. adaptation through microevolution). In the former, selection on the trait would remain unchanged if all individuals respond appropriately and to the same degree, whereas in the latter, selection would increase. We also distinguish whether the direction and/or the pace of the response is adaptive. A population advancing their phenology, but to an insufficient degree so as to keep pace with their primary food source, for example, would be considered to have a direction of response that is adaptive, but a pace that is not. A fully adaptive response would have both a sufficient direction and pace. Of the 12 studies on live mammals described above, all but three attempted to determine the fitness consequences of the observed phenotypic changes but in most cases inference was based on common-sense reasoning and individual fitness was not actually quantified (Table 1). No studies used reciprocal transplants, genotypic selection estimates or Q_ST_-F_ST_ comparisons as recommended by Merilä and Hendry (2014). Four studies found strong evidence that the phenotypic changes were not adaptive (i.e. changes in the trait were likely to be associated with a decline in fitness, see Fig. 3 for an example). In Columbian ground squirrels, individual fitness declined with the delayed emergences brought on by later snowmelt (Lane et al. 2012). In alpine marmots, female body size and litter size has decreased with decreased snow depth in winter (Tafani et al. 2013). In polar bears, the evidence points to body condition decreasing because of poor hunting conditions for the bears and this, in turn, has led to decreased body condition and survival and declining populations (Stirling and Derocher 2012). In the case of the Chillingham cattle, advanced breeding led to more calves being born in winter and calves born in winter suffered higher mortality (Burthe et al. 2011).

Selection favours early reproduction in female red squirrels (Réale et al. 2003a) and red deer (Coulson et al. 2003), and early antler growth in male red deer (Clements et al. 2010). However, Moyes et al. (2011) did not find any association between earlier breeding brought on by climate change and measures of reproductive success in either male or female red deer (Fig. 1). Similarly, for caribou, the direction of the observed phenological shift (i.e. an advance in timing of births) is possibly adaptive in that it is in the same direction as earlier plant growth (Post and Forchhammer 2008). The pace, however, is not as the response has been insufficient to remain in synchrony with timing of plant growth and calf recruitment was negatively correlated with the degree of mismatch between parturition date and plant phenology (Post and Forchhammer 2008). For yellow-bellied marmots, while there has been large advances in emergence date (23 days), breeding date (as measured by weaning dates) advanced by 5.6 days only (Ozgul et al. 2010). An extended growing season, brought on by earlier emergence from hibernation, has led to young-of-the-year yellow-bellied marmots entering hibernation in better condition (Ozgul et al. 2010). This, in turn, has led to increased over-winter survival and fitness and a threefold increase in population size. In this case, increasing body mass in response to climate change can be considered an adaptive response. For Soay sheep, whereas, selection favours larger body mass (Milner et al. 1999), there has been a temporal trend for decreasing size as ecological conditions promoting slower growth have overridden an evolutionary response for larger growth (Ozgul et al. 2009). Selection has thus been relaxed but fitness has been maintained so decreasing body masses can be considered adaptive here. In general, whether phenotypic responses in body mass to climate change are adaptive will often be difficult to interpret. Whereas larger bodied individuals often experience higher relative fitness (Kingsolver et al. 2001), according to one potential mechanism underlying Bergmann's rule (allometric scaling leading to higher themoregulatory efficiency in larger bodied individuals), body mass should decline in locations where climate change leads to warming temperatures (Teplitsky and Millien 2014). Better fasting endurance in larger bodied individuals (Lindstedt and Boyce 1985) however could lead to selection favouring large body size if climate change results in increasing climatic variability (Goodman et al. 2012).

## Evolutionary versus plastic responses

Our final objective was to examine whether any of the studies reporting phenotypic responses to climate change were able to reliably distinguish the underlying roles of phenotypic plasticity versus microevolution. To meet the strict definition of phenotypic plasticity, a study must demonstrate *individual* (or genotypic) variation in focal traits (de Jong 1995). As clonal mammals do not exist naturally, this requires the monitoring of individual phenotypes in different environments (e.g. across >1 year, termed individual plasticity in nature by Merilä and Hendry (2014). Observations of between-population variation in traits or cross-sectional analyses of populations across time do not meet the strict definition. For example, arctic ground squirrels (*Urocitellus paryii*) have been observed to differ in hibernation and reproductive phenology between two populations exposed to different weather conditions (Sherriff et al. 2011). While phenotypic plasticity in these traits is plausible, as the same individuals were not measured in the two populations, it has not been demonstrated. Similarly, while population-level temporal variation is often attributed to phenotypic plasticity, without assessing levels of individual variation, this will be difficult to conclude over the alternatives (e.g. microevolution). However, Merilä and Hendry (2014) provide a qualified argument that “fine-grained population responses” (year to year variation in environmental variable correlated to year-to-year variation in trait) could be used as a measure the importance of plasticity because genetic changes cannot track year-to-year variation, at least in species whose generation time is >1 year.

### Individual plasticity and fine-grained population responses

Although the majority of the studies we examined inferred evidence for plastic responses via fine-grained population responses relatively few have conclusively demonstrated individual plasticity in response to climate change. Parturition dates are phenotypically plastic in American red squirrels (Réale et al. 2003a,b) and individual Columbian ground squirrels (Lane et al. 2012) terminate hibernation earlier in response to warmer spring temperatures. All six phenological traits of red deer studied by Moyes et al. (2011) plastically advance in response to warmer springs. Weights of red deer calves produced by individual females (Nussey et al. 2005) are also heavier following warm springs. Interestingly, however, this effect was only observed in females experiencing favourable environmental conditions, highlighting the importance of evaluating phenotypic plasticity at the individual level. In Soay sheep and yellow-bellied marmots, body mass is plastic and has exhibited a consistent change over time (Ozgul et al. 2009, 2010).

### Quantitative genetic analyses

For a compelling demonstration of a microevolutionary response to climate change, a study must show that the trait in question is under selection, heritable, and has undergone change at a genetic level in response to temporal variation in the environment (Gienapp et al. 2008). Three studies provided the necessary information to demonstrate such a response (Table 1). (see *Response to Selection* below). In addition, evolutionary potential (i.e. underlying genetic variance) for a few additional traits and species has been inferred. The most powerful and flexible way to estimate genetic influences on phenotypic traits in wild, unmanipulated, populations is with a specific type of mixed-effects model, the animal model (Kruuk 2004). In the animal model, measures of genetic relatedness (usually estimated with a pedigree) and phenotypic similarity among individuals are compared to infer the variance components underlying observed phenotypic variation (see Kruuk 2004 for a detailed description of the animal model methodology). The requirement of a multi-generational pedigree has led to heritability estimates of traits likely to be associated with climate change having only been obtained from a select number of mammalian populations (Table 2). Parturition date is heritable in American red squirrels (Réale et al. 2003a,b), Columbian ground squirrels (Lane et al. 2011), red deer (Clements et al. 2010) and Soay sheep (Kruuk and Hadfield 2007). Body mass is heritable in big-horned sheep (*Ovis canadensis*; Réale et al. 1999), Soay sheep (Milner et al. 2000), leaf-eared mice (*Phylottis darwinii*; Nespolo et al. 2003) and weasels (*Mustela nivalis*; Zub et al. 2012). Energetic traits (e.g. basal metabolic rate) are heritable in at least one wild mammal [weasels (Zub et al. 2012)]. We include energetic traits here because, although little studied, they could have important ramifications both for the adaptation of wild mammal populations to their environment and the predictive ability of species distribution models (*sensu* Humphries et al. 2002). With respect to the former, mammalian metabolic rates exhibit significant variation across zoogeographical zones, potentially due to differences in mean climatic variables or climatic variability (Lovegrove 2000). As climate change could lead to analogous temporal changes, evolution of metabolic rates is possible.

**Table 2:** Published heritability and selection estimates for traits with putative associations to climate change in wild mammals. Error estimates (standard errors [SE], 95% highest posterior density intervals [HPD] or posterior standard deviations ([SD]) are included when presented in the original source

Species	Trait	*h*^*2*^	Selection gradient (*β*) and/or differential (*S*_*i*_)	References
American red squirrel (*Tamiasciurus hudsonicus*)	Parturition date	0.16 ± 0.03 (SE)***	*S*_*i*_ = −0.17 ± 0.05 (SE) ***	Réale et al. (2003a,b)
Columbian ground squirrel (*Urocitellus columbianus*)	Male emergence date from hibernation	0.22 ± 0.16 (SE) *	NA	Lane et al. (2011, 2012)
	Female emergence date from hibernation	0.22 ± 0.05 (SE) ***	*β* = −0.03 (−0.08 to 0.02; HPD)	
	Male body mass at emergence from hibernation	0.02 ± 0.15 (SE)	NA	
	Female body mass at emergence from hibernation	0.23 ± 0.09 (SE) ***	NA	
	Female oestrous date	0.18 ± 0.12 (SE)	NA	
Red deer (*Cervus elaphus*)	Male antler cast date	0.13 ± 0.08 (SE)	−0.03 ± 0.01 (SD) †	Clements et al. (2010, 2011); Coulson et al. (2003)
	Male antler clean date	0.15 ± 0.09 (SE)	−0.04 ± 0.02 (SD)	
	Male rut start date	0.26 ± 0.06 (SE) ***	NA	
	Male rut end date	0.17 ± 0.06 (SE) ***	NA	
	Female oestrous date	0.05 ± 0.04 (SE)	NA	
	Female parturition date	0.09 ± 0.03 (SE) ***	*β* = −0.65‡	
Soa sheep (*Ovis aries*)	Male body mass	0.12 ± 0.05 (SE)*	*β* = −0.25 ± 0.22 (SE) to 0.21 ± 0.16 (SE)§	Milner et al. (1999, 2000); Kruuk and Hadfield (2007)
	Female body mass	0.24 ± 0.09 (SE)*	*β* = −0.02 ± 0.05 (SE) to 0.17 ± 0.07 (SE)§	
	Parturition date	0.19***	NA	
Bighorn sheep (*Ovis canadensis*)	Body mass at maturity	0.03 ± 0.11 (SE) to 0.81 ± 0.09 (SE)***,¶ (both sexes pooled)	−0.21 ± 0.44 (SE) (male) and −0.05 ± 0.44 (SE) (female)	Réale et al. (1999); Coltman et al. (2005)
Leaf-eared mouse (*Phyllotis darwini*)	Basal metabolic rate	0.01 (warm) and 0.03 (cold)	NA	Nespolo et al. (2003)††
	Thermal conductance	0.05 (warm) and 0.66 (cold) *	NA	
	Body temperature	0.40 (day) and 0.68 (night)	NA	
	Body mass at sexual maturity	0.01	NA	
Weasel (*Mustela nivalis*)	Male body mass	0.61 ± 0.21 (SE) **	NA	Zub et al. (2012)
	Female body mass	0.32 ± 0.39 (SE)	NA	
	Mass corrected resting metabolic rate	0.45 ± 0.25 (SE) ***	NA	

* *P* < 0.05.

** *P* < 0.01.

*** *P* < 0.001.

† Statistically significant as inferred by confidence intervals that do not overlap with 0.

‡ Statistical significance not indicated in the original source.

§ Varies across years.

¶ Varies according to age and season.

†† Heritabilities for additional morphological and physiological traits are also provided by Nespolo et al. (2003). We report here the traits we deemed most relevant to climate change. Heritabilities of basal metabolic rate and thermal conductance were measured in both warm- and cold-acclimated animals and heritabilities of body temperature were measured during the day and night.

Selection is unlikely to act on phenotypic traits in isolation and, should genetically correlated traits be exposed to antagonistic selection pressures, rates of microevolution will be slowed (conversely, should they be exposed to synergistic selection pressures, rates will be accelerated). Through a comprehensive multivariate analysis of phenological traits within and between the sexes in red deer, Clements et al. (2010) showed that phenotypic correlations among phenological traits within a year were often strong, but these associations were not underlain by strong genetic correlations. In contrast, in Columbian ground squirrels there is a between-sex genetic correlation (*r*_*G*_ = 0.76 ± 0.22) in the date of termination of hibernation and female parturition date is strongly genetically correlated with hibernation termination date (*r*_*G*_ = 0.98 ± 0.01; Lane et al. 2011). At present, however, sufficient information is not available to produce a general consensus on how climate change will influence suites of life history traits.

### Response to selection

Microevolution, by definition, represents genetic change across generations (Futuyma 2009). Longitudinal studies of pedigreed populations can thus infer microevolution as changes in the genetic merit of traits (i.e. breeding values) across multiple generations. Attempts to do so, using best linear unbiased predictors (BLUPs; Henderson 1950, 1976) have been made in wild populations, but mammals have been rarely represented. One exception is the report of advancing phenologies in American red squirrels (Réale et al. 2003a). Parturition date is both heritable and under selection in this population (Réale et al. 2003b). The breeder's equation (i.e. R = h^2^S, where R represents the response to selection, h^2^ represents the heritability of the trait and S represents the selection differential acting on the trait; Falconer and Mackay 1996) thus predicts an evolutionary response for earlier parturition (predicted response = 0.6 days generation^−1^). This prediction corresponds well with the observed change in estimated breeding values (observed response = 0.8 days generation^−1^). Admittedly, criticisms regarding the use of BLUPs to estimate breeding values have been voiced recently (Postma 2006; Hadfield et al. 2010) and a more conservative analysis (a Bayesian posterior predictive test) has been advocated by Hadfield et al. (2010). We have recently reanalysed our data on American red squirrels and confirmed this. Using the posterior predictive test, the estimate of evolutionary change was similar to the previous account (R = 0.65 days generation^−1^). However, the Bayesian probability of this change being >0 is 0.75 (J. E. Lane and S. Boutin, unpublished manuscript), as compared to a previously reported *P* value of 0.001 (Réale et al. 2003a).

Ozgul et al. (2009, 2010) used the Price Equation to assess the role of micorevolution in changes in body mass of Soay sheep and yellow-bellied marmots. Although they were able to detect a small degree of micro-evolution in each case, the ecological relevance of this effect was considerably less important than plasticity.

### Experimental approaches

Three of the approaches for distinguishing phenotypic plasticity from genetic change in observations of phenotypic responses to climate change outlined by Merilä and Hendry (2014) are experimental. Common garden studies and space-for-time substitutions involve translocating individuals to environments in which they did not evolve or develop. In the third approach (experimental evolution), replicate populations are monitored under experimentally altered environments (e.g. elevated temperatures). Relatively long generation times are likely to render the latter approach infeasible for most mammalian species (but see below), but the former two have potential.

Common garden, reciprocal translocation and assisted migration studies could all be used to experimentally test for genetic adaptation to the environment and, by taking advantage of space-for-time substitutions, to infer potential genetic responses to climate change. In a common garden study, individuals from separate populations are raised under identical conditions in either a laboratory or natural setting (Conover and Schultz 1995). In so doing, environmental sources of variation are controlled for and observed phenotypic differences are thus inferred to represent genetic differences. In a reciprocal translocation study, individuals are moved between two populations exhibiting phenotypic variation. If appropriate controls are used (i.e. moving individuals within each population), this experimental design essentially provides two parallel common garden studies, but with the added ability to assess nonlinear responses (Endler 1986; Kawecki and Ebert 2004; Hereford 2009). Assisted migration *sensu stricto* refers to a conservation strategy of relocating individuals of threatened species outside of their natal range to areas projected to become suitable in the future as a result of climate change (McLachlan et al. 2007). While the ethical ramifications of such a strategy are hotly debated (Ricciardi and Simberloff 2009), their scientific merits are rarely discussed and almost entirely unknown. We are unaware of any published studies that have performed such an experimental approach with mammals and the logistics of doing so are likely to be formidable. However, interpopulation phenotypic variation both represents important raw material for adaptation to climate change (e.g. through the migration of warmer adapted species to higher elevations/latitudes) and also creates the opportunity for such an approach.

### Experimental evolution

Potential evolutionary responses to climate change can also be inferred through either artificial selection or experimental evolution. In both approaches, selection is implemented by the researcher (Conner 2003). In the former, the researcher directly selects on the trait of interest whilet in the latter, the researcher attempts to alter the selective landscape by manipulating one or more environmental variables. We are unaware of any studies that have used these approaches in mammals and their effectiveness is questionable given the relatively long generation times of mammals (see Collins et al. 2014 for examples from shorter generation taxa). One approach may be to take advantage of on-going experiments. For example, experimental forest plots have been artificially warmed for the past twenty years as part of the Harvard Forest soil warming experiment (Frey et al. 2013). Such an, in progress, experimental set-up could potentially facilitate study of higher trophic levels, including mammals.

### Genomics

The advent of cost-effective, high-resolution genome sequencing and genotyping has created the potential for researchers to document genetic responses to climate change directly (Stapleton et al. 2010). Previously, restricted to humans and species of economic relevance, researchers working on nonmodel species can now sequence genomes at 1000s of locations and genotype every individual in their population. A number of studies of wild mammals are embarking on such directions, but the challenges of using genomics to document microevolution in response to climate change are not insignificant. First, shifts in allele frequency at any locus arguably meet the definition of microevolution. As selection acts on phenotypes (and not genotypes), however, a more satisfying result would be to show allelic change at regions associated with variation in phenotypic traits responding to climate change. Identifying allelic variation at regions within candidate genes (e.g. the *Clock* gene; King et al. 1997) or association mapping for quantitative trait loci should thus be one objective. As well, genetic samples need to be collected over a sufficiently long duration to observe climate change-related variation. While many long-term studies will have archived tissue samples, definitive results from a newly initiated study should not be expected for a decade or more.

## Conclusions and future directions

**Figure 4:**
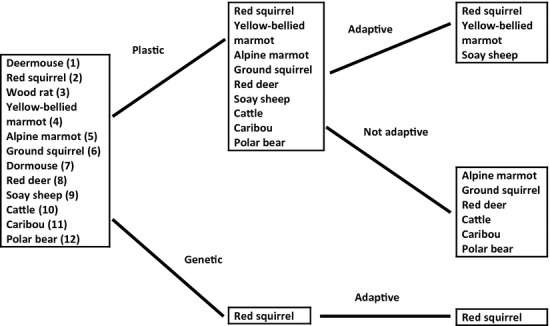
Summary of studies examining the link between climate change and plastic or evolutionary changes in traits in mammals. Of the 12 studies we found in the literature, nine found some evidence for a plastic response, however, in six cases the response was not adaptive. Four studies found some evidence that the responses were adaptive, but only one of these (red squirrels) had direct evidence for some of the phenotypic change being due to micro-evolution. Numbers next to each species correspond to references found in Table 1.

The results of our review are summarized in Fig. 4 with the key take-home being that current evidence for microevolution of mammal populations in response to recent climate change is negligible. Of the 12 studies we found in the literature, nine found some evidence for a plastic response; however, in six cases the response was likely not adaptive. Four studies found some evidence that the phenotypic changes were adaptive but only one of these (red squirrels) had direct evidence for some of the observed change being due to microevolution. Further analyses, however, suggest that these changes were not due to climate change. Unfortunately, it is too early to tell if our overall findings to date are due to the paucity of data or to the lack of evolution *per se*. The information required to provide definitive tests for the role of phenotypic plasticity versus evolution remains exceedingly rare for mammals. Our findings are consistent with the lack of evidence of evolutionary responses to climate change in avian phenology (Charmantier and Gienapp 2014) and other taxal groups (Reusch 2014; Schilthuizen and Kellermann 2014; Urban et al. 2014).

Not surprisingly, studies of mammals to date have been opportunistic in nature, reporting phenology-associated changes from long-term studies of populations covering more than two decades. The causal linkage to climate change has been correlative or “common sense” in nature but we caution that such approaches can lead to surprises. In our studies of American red squirrels, we found a tight correlation between cone supply and parturition date and used a common sense argument to link warmer, drier springs to higher cone crops and therefore earlier breeding. The striking pattern of advancement of breeding over the first 10 years of the study completely reversed in the subsequent 10 years and although our initial conclusion that both phenotypic plasticity and evolution were involved has held true, the link to climate change seems unlikely (Krebs et al. 2012).

Given that the evolution shown in red squirrels was not linked to climate change, we conclude that all adaptive phenotypic changes in response to climate change reported for mammals to date, appear to primarily be due to phenotypic plasticity. This trend may change however, as climate change pushes environmental conditions beyond recent year-to-year variation for which phenotypic plasticity has evolved. It is entirely likely that more decades of monitoring will be required before we have an answer.

Merilä and Hendry (2014) provide a clear outline of the challenges that longitudinal studies must meet to address the role of phenotypic plasticity versus evolution in climate-driven phenotypic changes. Given these challenges, few studies are likely to be successful and although long-term individual-based studies (complete with genetic pedigrees) will continue to provide insight into evolutionary versus plastic change in mammals, there is a real need to compliment these with experimental and/or genomics studies. Clearly, major challenges exist in creating artificial climates; however, there is also a long record of studies using small mammals in semi-natural enclosures or on islands that suggest experimental transplants are possible. Similarly, genomics techniques have yet to be applied to address the influences of climate change on wild mammals but the rapid development of this field, coupled with the availability of archived DNA from long-term studies, should present this opportunity in the near future.
